# Supplementary material for: Does host-guest interaction promote tolerance behavior? The mediating role of place attachment and subjective well-being
Source: PLoS One. 2025 May 28;20(5):e0323355. doi: 10.1371/journal.pone.0323355 (PMC12118835; doi:10.1371/journal.pone.0323355)
Supplement: Attachment — Submitted filename: Response to Reviewers.docx [file pone.0323355.s002.docx]

**Response Letter**

**Dear** **Editors and Reviewers:**

Firstly, I would like to extend my sincere gratitude to you and the reviewers for the invaluable feedback and suggestions provided on my previous submission. Your comments have been instrumental not only in advancing the improvement of my research but also in significantly enhancing the overall quality of the manuscript.

Following a thorough revision process, I have comprehensively addressed each suggestion put forth by both the editorial office and reviewers. I am confident that these enhancements have substantially improved the content, logic, and clarity of the manuscript, aligning it more closely with your publication standards.

Should there be any additional areas for refinement, I will greatly appreciate your insights and strive diligently to further enhance the manuscript. Once again, I express my profound appreciation for your dedicated efforts and professional guidance. I eagerly await your positive response!

Yours sincerely,

Ke Wu

**The Editors**

We sincerely appreciate the time you dedicated to reviewing our manuscript and for offering invaluable feedback and suggestions regarding our research. We would like to extend our heartfelt gratitude to you.

In response to the points you've raised, we will address each one individually:

**Question1:**  Please ensure that your manuscript meets PLOS ONE's style requirements, including those for file naming. The PLOS ONE style templates can be foundathttps://journals.plos.org/plosone/s/file?id=wjVg/PLOSOne_formatting_sample_main_body.pdfandhttps://journals.plos.org/plosone/s/file?id=ba62/PLOSOne_formatting_sample_title_authors_affiliations.pdf

**Response:** I sincerely appreciate your reminder regarding the requirements for PLOS ONE manuscripts, particularly concerning the file naming conventions. I have meticulously reviewed the submission guidelines for PLOS ONE and have diligently checked and adjusted my manuscript to ensure that its content, format, and file naming adhere strictly to the standards set forth by your journal.

**Question2:** Thank you for stating in your Funding Statement: "This research was supported by The National Natural Science Foundation of China project “Research on the Impact of Social Network of Ethnic Village Farmers on Tourism Livelihood Behavior” (72064006). Institute of Guangxi Tourism Industry Scientiffc Research Fund: Research on the Inffuencing Factors of Cultural Capital of Ethnic Village Farmers on Community Citizen Behavior (LYCY2023-29);Evaluation of Livelihood Resilience of Ethnic Tourism Villages and Its Driving Mechanism(LYCY2023-06). "Please provide an amended statement that declares *all* the funding or sources of support (whether external or internal to your organization) received during this study, as detailed online in our guide for authors at http://journals.plos.org/plosone/s/submit-now. Please also include the statement “There was no additional external funding received for this study.” in your updated Funding Statement. Please include your amended Funding Statement within your cover letter. We will change the online submission form on your behalf.

**Response:** I would like to extend my heartfelt appreciation for your attention to this study and your invaluable guidance regarding the specifics of the funding statement. In accordance with your request, I have undertaken a thorough revision of the funding statement to ensure it comprehensively addresses all sources of funding and support received throughout this study while adhering to the online author guidelines set forth by your journal. Below is the revised funding statement:

This research was supported by the National Natural Science Foundation of China [Grant No. 72064006, 72462013].

**Question3:** We note that your Data Availability Statement is currently as follows: All relevant data are within the manuscript and its Supporting Information files.Please confirm at this time whether or not your submission contains all raw data required to replicate the results of your study. Authors must share the “minimal data set” for their submission. PLOS defines the minimal data set to consist of the data required to replicate all study findings reported in the article, as well as related metadata and methods(https://journals.plos.org/plosone/s/data-availability#loc-minimal-data-set-definition).For example, authors should submit the following data:

- The values behind the means, standard deviations and other measures reported;
- The values used to build graphs;
- The points extracted from images for analysis.Authors do not need to submit their entire data set if only a portion of the data was used in the reported study.If your submission does not contain these data, please either upload them as Supporting Information files or deposit them to a stable, public repository and provide us with the relevant URLs, DOIs, or accession numbers. For a list of recommended repositories, please see https://journals.plos.org/plosone/s/recommended-repositories.If there are ethical or legal restrictions on sharing a de-identified data set, please explain them in detail (e.g., data contain potentially sensitive information, data are owned by a third-party organization, etc.) and who has imposed them (e.g., an ethics committee). Please also provide contact information for a data access committee, ethics committee, or other institutional body to which data requests may be sent. If data are owned by a third party, please indicate how others may request data access.

**Response:** We sincerely appreciate your comprehensive review and insightful feedback regarding our research. The findings we presented are derived from a subset of the data, which has been provided as supplementary information files. Kindly refer to the attached documents for further details.

**Question4:** PLOS requires an ORCID iD for the corresponding author in Editorial Manager on papers submitted after December 6th, 2016. Please ensure that you have an ORCID iD and that it is validated in Editorial Manager. To do this, go to ‘Update my Information’ (in the upper left-hand corner of the main menu), and click on the Fetch/Validate link next to the ORCID field. This will take you to the ORCID site and allow you to create a new iD or authenticate a pre-existing iD in Editorial Manager.

**Response:** I sincerely appreciate your comprehensive review and insightful feedback regarding our research. In accordance with PLOS requirements, I have confirmed that I, as the corresponding author, possess a valid ORCID iD, which has been duly verified in Editorial Manager.

**Question5:** Your ethics statement should only appear in the Methods section of your manuscript. If your ethics statement is written in any section besides the Methods, please delete it from any other section. 
**Response:** I would like to extend my heartfelt appreciation for your comprehensive review and insightful feedback on our study. I have meticulously examined the manuscript and confirmed that the ethical statement has been retained solely in the 'Methods' section. Additionally, I have eliminated all pertinent content related to the ethical statement from other sections of the manuscript to ensure both compliance and clarity.

**Reviewer: 1**

We would like to express our sincere gratitude to the reviewers for dedicating their valuable time amidst their busy schedules to evaluate our manuscript. The constructive suggestions provided by the reviewers significantly enhance the quality of our work. We extend our heartfelt appreciation for your efforts.

In response to all the points you have raised, we will address them individually as follows:

**Question 1：**I regret to inform you that I cannot recommend this manuscript for publication. The overall writing quality does not meet the standards expected for academic work, which affects the clarity and professionalism of the paper.

**Response:** We sincerely appreciate your comprehensive review and invaluable feedback on our study. In response to your earlier comment, 'The overall writing quality does not meet the standards expected for academic work, which affects the clarity and professionalism of the paper,' we have engaged in a thorough self-assessment and fully acknowledge your critique. Consequently, we have meticulously revised the sentence structure, grammar, and spelling throughout the manuscript to ensure both accuracy and fluency in our content. These enhancements are intended to improve the article's readability while effectively conveying our research findings.

**Question 2:** Additionally, there is a clear disconnect between the introduction and the literature review, with the latter failing to adequately explain the relationships between the variables under investigation.

**Response:** We sincerely appreciate your comprehensive review and invaluable feedback on our study. In response to the concern you raised that " there is a clear disconnect between the introduction and the literature review, with the latter failing to adequately explain the relationships between the variables under investigation", we have implemented targeted revisions. We have restructured the logical framework of the **Introduction** by first **outlining the academic context of tolerance behavior**, followed by **a discussion of the current research landscape of tolerance behavior, an analyse of host-guest interaction dynamics, an exploration of place attachment and subjective well-being as mediating mechanisms**, and **a** **summary of the theoretical contributions of this paper**". Additionally, we have eliminated redundant content to more effectively clarify these relationships among research variables. The supplementary content is detailed below:

Tolerance, as a customer behavior, is increasingly receiving widespread attention from academia[1]. Specifically, tolerance refers to the degree of forgiveness displayed by customers when service delivery fails to meet their expectations[1]. This concept emphasizes the patient and forgiving attitude of customers in service contexts[2]. Tolerance behavior not only helps customers achieve inner balance, but also provides valuable opportunities for service providers to repair potential cracks in customer relationships caused by service deficiencies[3]. Especially in the tourism industry, tolerance behavior, as a manifestation of tourist citizenship behavior [4], is of great significance for promoting the long-term stable development of the tourism industry and enhancing competitive advantages [5, 6]. Therefore, the in-depth study of TTB by academia not only enriches customer behavior theory but also provides new perspectives and strategic ideas for enhancing tourism service quality.

As TTB has increasingly emerged as a focal point of academic inquiry, scholars have investigated the multiple factors influencing TTB from diverse perspectives. These factors can primarily be divided into two dimensions: organizational and individual factors. In terms of organizational factors, research encompasses critical elements such as rule-making[7] and hotel room pricing[8]. In terms of individual factors, it addresses customers' negative emotions[9] and their perceptions of climate change [10]. However, the above research context on TTB is mainly limited to the hotel industry[7-9], given that hotels represent relatively enclosed environments where guest interactions with staff constitute the primary service engagement. Conversely, tourist attractions are characterized by more open settings in which tourists engage with guides, area personnel, and local residents in varied ways. Consequently, findings related to tolerant behavior within hotel contexts cannot be directly extrapolated to scenarios involving tourist destinations [7-9]. Furthermore, interactions between tourists and local residents at these destinations are recognized as pivotal for fostering long-term sustainable development in tourism areas[11]. Previous studies have shown that interactive experiences within tourist attractions have a significant impact on tourists' behavior, such as stimulating their participation enthusiasm[12] and promoting word-of-mouth recommendations and the formation of sustained usage intentions[13]. Therefore, it is crucial to thoroughly investigate whether—and how—the interaction between tourists and local residents in open tourist attractions impacts TTB.

To further investigate the mechanisms underlying TTB, this study introduces two psychological variables—place attachment (PA) and subjective well-being (SWB)—as significant mediators. The PA theory posits that as individuals strengthen their emotional connections to a specific location, their perceptions and behaviors towards tourism destinations are profoundly affected (14). Empirical research has demonstrated that tourists' sense of place identity and positive emotions can enhance pro-environmental behaviors (15, 16), while feelings of awe toward tourism sites directly foster helping behaviors (17). Consequently, this study seeks to elucidate the intrinsic relationship between HGI and TTB through the mediating role of PA. Additionally, drawing from emotion regulation theory, individual SWB exerts a substantial positive influence on behavior (18). In workplace contexts, SWB significantly enhances organizational citizenship behavior (18) and also impacts tourists' decision-making processes regarding travel (19). Despite numerous valuable studies examining tourists' PA emotions and SWB within academic discourse, there remains a paucity of research integrating both constructs to uncover the fundamental drivers behind tourist behavior. Therefore, this study aims to explore in greater depth and comprehensiveness the intrinsic connection between HGI and TTB via the mediating variables of PA and SWB.

The literature contribution of this study to TTB is mainly reflected in the following aspects: Firstly, it underscores the critical role of HGI in shaping TTB within tourist destinations. By establishing HGI as a fundamental prerequisite for TTB, this research addresses whether tourists who engage more interactively are indeed more tolerant. Secondly, it elucidates the underlying mechanisms through which HGI influences TTB by incorporating PA and SWB. Lastly, this investigation is situated within the context of open tourist attractions, thereby broadening the scope of research on tolerance behavior in tourism and offering a theoretical framework for exploring analogous relationships in related fields. Consequently, this study paves new avenues for future inquiries into civic behavior among tourists. More broadly, it provides essential theoretical support and practical insights aimed at enhancing TTB in tourist destinations.

The references are as follows：

1. *Yi Y, Gong T. Customer value co-creation behavior: Scale development and validation. J Bus Res. 2013;66(9):1279-84.*
2. *Yang Y, Hu J. Self-diminishing effects of awe on consumer forgiveness in service encounters. Journal of Retailing and Consumer Services. 2021;60.*
3. *Hur J, Jang S. Is consumer forgiveness possible? International Journal of Contemporary Hospitality Management. 2019;31(4):1567-87.*
4. *Zhang H, Xu H. Impact of destination psychological ownership on residents’ “place citizenship behavior”. J Destin Mark Manage. 2019;14.*
5. *Torres-Moraga E, Rodriguez-Sanchez C, Sancho-Esper F. Understanding tourist citizenship behavior at the destination level. J Hosp Tour Manag. 2021;49:592-600.*
6. *Yao Y, Wang G, Ren L, Qiu H. Exploring tourist citizenship behavior in wellness tourism destinations: The role of recovery perception and psychological ownership. J Hosp Tour Manag. 2023;55:209-19.*
7. *Ma Shuang LX, Li Chunqing. Customer citizenship Behavior and misbehavior in the Context of Sharing economy: from the perspective of Social Dilemma Theory. Advances in psychological science. 2022;29(11):1920-35.*
8. *Alderighi M, Nava CR, Calabrese M, Christille J-M, Salvemini CB. Consumer perception of price fairness and dynamic pricing: Evidence from Booking.com. J Bus Res. 2022;145:769-83.*
9. *Xiong Wei HM, Huang Yuanfei. The elastic change of customer's negative emotion and tolerance in the context of hotel service failure tourism science. 2021;35:53-75.*
10. *Seekamp E, Jurjonas M, Bitsura-Meszaros K. Influences on coastal tourism demand and substitution behaviors from climate change impacts and hazard recovery responses. J Sustain Tour. 2019;27(5):629-48.*
11. *Eusebio C, Vieira AL, Lima S. Place attachment, host-tourist interactions, and residents' attitudes towards tourism development: the case of Boa Vista Island in Cape Verde. J Sustain Tour. 2018;26(6):890-909.*
12. *Wei MQ, Bai CH, Li CX, Wang HY. The effect of host-guest interaction in tourist co-creation in public services: evidence from Hangzhou. Asia Pac J Tour Res. 2020;25(4):457-72.*
13. *Moon H, Miao L, Hanks L, Line ND. Peer-to-peer interactions: Perspectives of Airbnb guests and hosts. International Journal of Hospitality Management. 2019;77:405-14.*
14. *Jin MLC, Y.Lee, C. K.Ahmad, M. S. Effects of Place Attachment and Image on Revisit Intention in an Ecotourism Destination: Using an Extended Model of Goal-Directed Behavior. Sustainability-Basel. 2020;12(18).*
15. *Gezhi C, Xiang H. From good feelings to good behavior: Exploring the impacts of positive emotions on tourist environmentally responsible behavior. J Hosp Tour Manag. 2022;50:1-9.*
16. *Qu Y, Xu F, Lyu X. Motivational place attachment dimensions and the pro-environmental behaviour intention of mass tourists: a moderated mediation model. Curr Issues Tour. 2017;22(2):197-217.*
17. *Chen R, Bai K, Luo Q. To help or not to help: the effects of awe on residents and tourists’ helping behaviour in tourism. J Travel Tour Mark. 2021;38(7):682-95.*
18. *Xu J, Xie B, Chung B. Bridging the Gap between Affective Well-Being and Organizational Citizenship Behavior: The Role of Work Engagement and Collectivist Orientation. Int J Environ Res Public Health. 2019;16(22).*
19. *Skard SK, E. S., Sjastad HT, H. How virtual reality influences travel intentions: The role of mental imagery and happiness forecasting. Tourism Manage. 2021;87.*

In the meantime, we have meticulously revised the **Theoretical Basis and Hypothesis Proposal** sections to enhance the derivation process of our hypotheses, thereby ensuring that the hypothesis proposal is more logically coherent. Furthermore, we have comprehensively updated these sections by incorporating pertinent literature published between 2020 and 2024, which reflects the latest advancements and trends in our research domain. By thoroughly describing and analyzing these recent studies, we aim to provide a more holistic representation of the current state of research in this field while enriching both the depth and breadth of our study. Below is the supplementary content:

## The mediating role of PA

PA is not a static phenomenon; rather, it is dynamic and evolves as tourists engage with their travel destinations, gradually fostering a robust emotional connection[14]. According to the SOR theory, individuals are significantly influenced by external stimuli and environmental factors, resulting in corresponding behavioral responses[35]. As a crucial component of travel experiences, HGI is anticipated to enhance the experiential value for tourists and further reinforce their PA towards the destination [36]. For instance, Woosnam discovered that interactions between tourists and local residents at World Heritage sites contribute to mutual attachment to these locations [37]. Additionally, Wang et al. highlighted that interactions between tourists and service providers positively influence PA levels[38]. Consequently, this study posits that HGI can effectively stimulate and strengthen tourists' attachment to their travel destinations. Based on this premise, we propose the following hypothesis:

**H2).** HGI has a significant positive effect on PA .

The emotional attachment individuals have to a place can significantly shape the values and behaviors of social groups[39], with personal identification and a sense of belonging serving as essential prerequisites for individual action. Previous studies have confirmed that PA has a positive and significant impact on tourists' environmental responsibility behaviors[40], intentions[41], and willingness to return[42]. In addition, combined with the attitude-behavior theory, PA, as a profound human-place emotional connection, is regarded as a key psychological factor that has an important impact on tourists' behavior and decision making[43]. Based on previous studies, this study posits that PA positively influences TTB. Consequently, the following hypothesis is proposed:

**H3).** PA has a significant positive effect on TTB.

The SOR theory posits that external environmental stimuli can elicit emotional responses within individuals, which subsequently lead to the development of corresponding attitudes and ultimately influence behavioral responses. PA is a kind of spiritual bond generated during the interaction between tourists and tourist destinations[14]. When individuals have a higher positive emotion towards the tourist destination, they will show a strong behavioral inclinations[43]. Moreover, PA serves as a mediating factor in various relational dynamics, such as those between restorative sensory perception and environmentally responsible behavior[40], as well as gastronomic experiences and behavioral intentions[41]. These findings underscore the viability of PA as a mediator and its substantial impact on individual behavior. Consequently, we hypothesize that PA may evoke specific behavioral responses when stimulated by HGI. Based on this premise, we propose the following hypotheses:

**H4).** PA plays a mediating role between HGI and TTB.

## The mediating role of SWB

Positive interaction is an important driver of tourists' SWB [48]. A lack of social interaction can lead to a diminished sense of well-being[49]. Conversely, even minimal social engagements can significantly enhance SWB in everyday life[50], contributing to greater happiness among individuals[51, 52].Existing literature indicates that teacher-student interactions positively and significantly influence students' classroom SWB[53],HGI on residents' happiness[54, 55], and human interaction on tourists' SWB[56]. Moreover, HGI offers tourists diverse travel experiences[12], which in turn can affect their SWB[57]. Therefore, the interaction between tourists and residents may have an impact on the SWB of tourists. Based on this premise, we propose the following hypothesis:

**H5).** HGI has a significant positive effect on SWB.

The theory of emotion regulation in psychology posits that an individual's emotional state and experiences can significantly influence their behavior[18]. For instance, as women's sense of happiness increases, they may exhibit greater resilience to the pressures associated with economic and caregiving responsibilities[58]. Likewise, the SWB of tourists during their travels has a positive and substantial effect on their decision-making behaviors regarding travel [19], intentions to revisit [59], and overall loyalty [60]. Drawing from prior research, this study asserts that SWB is a critical factor affecting tourists' behavioral intentions. Contented tourists are more inclined to demonstrate tolerance in their actions. Consequently, we propose the following hypothesis:

**H6).** SWB has a significant positive effect on TTB.

The SOR theory posits that external environmental stimuli can elicit emotional responses within the body, which subsequently influence behavioral reactions. Moreover, SWB serves as a mediator in various relational dynamics, such as the connection between landscape appeal and intentions for repeat visits[59], as well as the association between nostalgic emotions and intentions for repeat visits[61]. These findings underscore the viability of SWB as a mediating factor and its substantial impact on individual behavior. Consequently, we propose that SWB, representing an individual's most authentic feelings, can be conceptualized as an organism's subjective experience. In response to HGI stimulation, this subjective experience may provoke corresponding behavioral reactions. Building upon this premise, we further advance the following hypothesis:

**H7).** SWB plays a mediating role between HGI and TTB.

The references are as follows：

1. *Wei MQ, Bai CH, Li CX, Wang HY. The effect of host-guest interaction in tourist co-creation in public services: evidence from Hangzhou. Asia Pac J Tour Res. 2020;25(4):457-72.*
2. *Jin MLC, Y.Lee, C. K.Ahmad, M. S. Effects of Place Attachment and Image on Revisit Intention in an Ecotourism Destination: Using an Extended Model of Goal-Directed Behavior. Sustainability-Basel. 2020;12(18).*
3. *Xu J, Xie B, Chung B. Bridging the Gap between Affective Well-Being and Organizational Citizenship Behavior: The Role of Work Engagement and Collectivist Orientation. Int J Environ Res Public Health. 2019;16(22).*
4. *Skard SK, E. S., Sjastad HT, H. How virtual reality influences travel intentions: The role of mental imagery and happiness forecasting. Tourism Manage. 2021;87.*
5. *Darley JM, & Gilbert, D. T. Social psychological aspects of environmental psychology. Handbook of social psychology. 1985;2:949–92.*
6. *Bi J, Wang B, Lu F. Does Host-Guest Interaction Stimulate Tourists’ Citizenship Behavior? A Combination of Social Exchange Theory and Cognitive Appraisal Theory. Forests. 2024;15(7).*
7. *Woosnam KM, Aleshinloye KD, Ribeiro MA, Stylidis D, Jiang J, Erul E. Social determinants of place attachment at a World Heritage Site. Tourism Manage. 2018;67:139-46.*
8. *Wang S, Lehto X, Cai LP, Behnke C, Kirillova K. Travelers' Psychological Comfort with Local Food Experiences and Place Attachment. J Hosp Tour Res. 2021.*
9. *D C. The facets of place. Springer: Boston,MA; 1997.*
10. *Wu K, Guo Y, Han X. The relationship research between restorative perception, local attachment and environmental responsible behavior of urban park recreationists. Heliyon. 2024;10(15).*
11. *Akoğul E, and Gökalp N. Selçuk. Effect of international food tourists’ food experiences on place attachment and behavioral intentions: The moderating role of food variety seeking tendency Journal of Vacation Marketing 2024:13567667241230345.*
12. *Pu B, Wei J, Zhou L, Li L. A study on the impact of tourism destination image and local attachment on the revisit intention: The moderating effect of perceived risk. Plos One. 2024;19(1).*
13. *Isa SM, Ariyanto HH, Kiumarsi S. The effect of place attachment on visitors' revisit intentions: evidence from Batam. Tourism Geogr. 2020;22(1):51-82.*
14. *Kim K, Uysal M, Sirgy MJ. How does tourism in a community impact the quality of life of community residents? Tourism Manage. 2013;36:527-40.*
15. *Woo E, Kim H, Uysal M. Life satisfaction and support for tourism development. Ann Tourism Res. 2015;50:84-97.*
16. *Johnson DCSaDM. Avowed happiness as an overall assessment of the quality of life. Social Indicators Research. 1978;5(4):475-92.*
17. *Durkin J, Joseph S. Growth Following Adversity and Its Relation with Subjective Well-Being and Psychological Well-Being. Journal of Loss and Trauma. 2009;14(3):228-34.*
18. *Su Lujun, He Xuehuan, Hu Dongbin. Research Progress and Implications of Tourists' Subjective Well-being [J]. Journal of Sichuan Normal University (Social Sciences Edition), 2019, 46(02): 83-92.*
19. *Baumeister RF, and Mark R. Leary. The need to belong desire for interpersonal attachments as a fundamental human motivation. Psychological bulletin. 1995;117(3):497.*
20. *Gunaydin G, Oztekin H, Karabulut DH, Salman-Engin S. Minimal Social Interactions with Strangers Predict Greater Subjective Well-Being. J Happiness Stud. 2021;22(4):1839-53.*
21. *Sandstrom GM, Dunn EW. Social Interactions and Well-Being: The Surprising Power of Weak Ties. Pers Soc Psychol Bull. 2014;40(7):910-22.*
22. *Kroencke L, Harari GM, Back MD, Wagner J. Well-being in social interactions: Examining personality-situation dynamics in face-to-face and computer-mediated communication. Journal of Personality and Social Psychology. 2023;124(2):437.*
23. *Xiao M, Tian Z, Xu W. Impact of teacher-student interaction on students’ classroom well-being under online education environment. Education and Information Technologies. 2023;28(11):14669-91.*
24. *Chen Y, Cottam E, Lin Z. The effect of resident-tourist value co-creation on residents’ well-being. J Hosp Tour Manag. 2020;44:30-7.*
25. *Wang H, Sha H, Liu L, Zhao H. Exploring the Relationship between Perceived Community Support and Psychological Well-Being of Tourist Destinations Residents. Int J Env Res Pub He. 2022;19(21).*
26. *van Esch P, Cui Y, Das G, Jain SP, Wirtz J. Tourists and AI: A political ideology perspective. Ann Tourism Res. 2022;97.*
27. *Yang S, Liu Y, Xu L. The effect of food tourism experiences on tourists' subjective well-being. Heliyon. 2024;10(3).*
28. *Ranganathan M, Stern E, Knight L, Muvhango L, Molebatsi M, Polzer-Ngwato T, et al. Women's economic status, male authority patterns and intimate partner violence: a qualitative study in rural North West Province, South Africa. Cult Health Sex. 2022;24(5):717-34.*
29. *Dat LT, Wu HC, Li TN, Huang WS, Liou GB, Hsieh CM. The effects of landscape fascination on subjective well‐being and revisit intention: Evidence from agritourism destinations. International Journal of Tourism Research. 2024;26(1).*
30. *Liu SP, Li SY, Chen YQ, Zheng TX. Examining relationships among food's perceived value, well-being, and tourists' loyalty. J Vacat Mark. 2023;29(2):161-74.*
31. *Karagöz D, and Haywantee Ramkissoon. Nostalgic emotions, meaning in life, subjective well-being and revisit intentions. Tourism Management Perspectives. 2023;48:101159.*

**Question 3:**The empirical analysis is not only poorly connected to the theoretical framework but is also overly simplistic, relying on basic validation without deeper exploration. Consequently, the discussion that follows is superficial and lacks substantive insights. Due to these significant shortcomings, I must recommend rejection of this manuscript.

**Response:** We sincerely appreciate your comprehensive review and invaluable feedback regarding our research. We take your critique, stating that ''the empirical analysis is not only inadequately linked to the theoretical framework but also overly simplistic, relying on basic validation without deeper exploration; as a result, the subsequent discussion is superficial and lacks substantive insights,'' with utmost seriousness and have engaged in thorough reflection. In response to this concern, we have meticulously re-evaluated and clarified our theoretical hypotheses while ensuring that the empirical analysis is closely connected to and deeply integrated with the theoretical framework, thereby enhancing the overall logical coherence and consistency of our study. Below is additional information:

This study investigated the interrelationships among HGI, PA, SWB, and TTB across two distinct tourist destinations by developing a conceptual model. The findings revealed that tourists' perceptions of their interactions with local residents positively influenced both their PA and SWB, thereby fostering TTB. Additionally, the research identified the specific mediating role of PA in the relationship between SWB and TTB. Through the collection and analysis of tourists' insights, this study not only advanced existing literature on tolerance behavior but also broadened the current research framework.

Firstly, we established that HGI exerts a significant positive influence on TTB. In contrast to prior studies that regarded HGI as either a prerequisite or moderating factor in resident behavior[28], this research specifically examines the impact of HGI on tourist behavior. The findings reveal that positive interactions can act as a catalyst, shaping tourists' behavioral responses. This aligns with earlier research indicating that HGI promotes tourist behavior[23, 26, 29]. By presenting new evidence, this study further substantiates the assertion that HGI can affect TTB.

Secondly,It is verified that PA plays a partial mediating role between HGI and TTB. On one hand, this suggests that interactions between tourists and hosts at destinations can enhance tourists' emotional connection to those locations. This conclusion aligns with previous research indicating that such interactions significantly influence tourists' cognition and emotions[37]; specifically, tourists exhibit corresponding PA in response to positive HGI, reflecting a favorable reaction towards it.On the other hand, PA serves as a direct precursor to TTB. An individual's emotional bond with a destination positively impacts their tolerance behaviors toward it, which corroborates existing literature asserting that tourists' emotional attachment and identification shape their attitudes towards destinations and subsequently affect their behaviors and decisions[43].

Thirdly, it has been established that SWB serves as a partial mediator between HGI and TTB. On one hand, prior research predominantly concentrated on the impact of HGI on residents' well-being[54, 55], whereas our study broadens the scope to include tourists at tourist destinations, thereby offering deeper insights into its positive effects on this demographic. Our findings suggest that HGI can stimulate SWB among tourists within the tourism context. This conclusion aligns with previous studies demonstrating significant positive correlations between HGI and tourists' SWB[50]. On the other hand, our research indicates that when tourists experience higher levels of happiness in these destinations, they are more inclined to exhibit understanding and tolerance. This outcome corroborates the emotion regulation theory's assertion that an individual's emotional state can significantly influence their behavior [18]. Specifically, elevated SWB among tourists is associated with increased positive emotions and psychological satisfaction, making them more likely to respond favorably even in adverse tourism situations.

The references are as follows：

1. *Xu J, Xie B, Chung B. Bridging the Gap between Affective Well-Being and Organizational Citizenship Behavior: The Role of Work Engagement and Collectivist Orientation. Int J Environ Res Public Health. 2019;16(22).*
2. *Tse S, Tung VWS. Measuring the Valence and Intensity of Residents' Behaviors in Host-Tourist Interactions: Implications for Destination Image and Destination Competitiveness. Journal of Travel Research. 2022;61(3):565-80.*
3. *Tu H, Ma J. Does Positive Contact Between Residents and Tourists Stimulate Tourists’ Environmentally Responsible Behavior? The Role of Gratitude and Boundary Conditions. Journal of Travel Research. 2021;61(8):1774-90.*
4. *Wang J, Yang X, Xi Y, He Z. Is Green Spread? The Spillover Effect of Community Green Interaction on Related Green Purchase Behavior. Int J Environ Res Public Health. 2022;19(11).*
5. *Li SH, Liu M, Wei M. Host sincerity and tourist environmentally responsible behavior: The mediating role of tourists' emotional solidarity with hosts. J Destin Mark Manage. 2021;19.*
6. *Woosnam KM, Aleshinloye KD, Ribeiro MA, Stylidis D, Jiang J, Erul E. Social determinants of place attachment at a World Heritage Site. Tourism Manage. 2018;67:139-46.*
7. *Isa SM, Ariyanto HH, Kiumarsi S. The effect of place attachment on visitors' revisit intentions: evidence from Batam. Tourism Geogr. 2020;22(1):51-82.*
8. *Gunaydin G, Oztekin H, Karabulut DH, Salman-Engin S. Minimal Social Interactions with Strangers Predict Greater Subjective Well-Being. J Happiness Stud. 2021;22(4):1839-53.*
9. *Chen Y, Cottam E, Lin Z. The effect of resident-tourist value co-creation on residents’ well-being. J Hosp Tour Manag. 2020;44:30-7.*
10. *Wang H, Sha H, Liu L, Zhao H. Exploring the Relationship between Perceived Community Support and Psychological Well-Being of Tourist Destinations Residents. Int J Env Res Pub He. 2022;19(21).*

To enhance your experience, we have marked the revised sections in blue within the text. We sincerely appreciate your invaluable suggestions and remain committed to elevating the quality of our research. Should there be any further opportunities for improvement, we would welcome your insights and will make every effort to implement them effectively. Thank you once again for your dedication and expert guidance.

**Reviewer: 2**

We would like to extend our sincere gratitude to you for the positive assessment and invaluable suggestions regarding our work. We have meticulously considered all comments and appropriately incorporated them into the revised manuscript. Here, we wish to convey our heartfelt appreciation to you.

In response to each of the points you raised, we will address them individually:

**Question 1：**First of all, it is recommended that the entire text undergo necessary language polishing. Currently, some grammar and expressions are not very academic. For example, the names of specific case locations should be capitalized, and it seems that the authors did not follow this rule in the abstract.

**Response:** We sincerely appreciate your meticulous review and invaluable feedback regarding our research. In response to your suggestions for language refinement, we have conducted a comprehensive review and revision of the entire manuscript. We have ensured that all place names adhere strictly to the appropriate capitalization conventions, while also meticulously examining and rectifying any potential grammatical inaccuracies or inappropriate expressions to enhance both the accuracy and fluency of the content. These enhancements are intended to improve the article's readability and effectively communicate our research findings.

**Question 2：**The logic of the introduction is not smooth. It is inappropriate for the author to start with tourist civic behavior as the background. It is recommended to start directly from the academic background and theoretical background of host-guest interaction or tourist tolerance.

**Response:** We sincerely appreciate your invaluable feedback on our study, particularly your insightful suggestion regarding the enhancement of the logical flow in the **introduction**. We wholeheartedly concur with your perspective and have implemented substantial revisions to this section. Specifically, we eliminated superfluous content and commenced with a comprehensive overview of the academic background surrounding tourism tolerance, elucidating its definition, current research landscape, and significance within the field of tourism. These modifications have strengthened the connection between our study's background and theoretical framework, thereby providing readers with a coherent research structure that clarifies the article's logic and facilitates a better understanding of our research topic and objectives. The revised **introduction** is as follows:

Tolerance, as a customer behavior, is increasingly receiving widespread attention from academia[1]. Specifically, tolerance refers to the degree of forgiveness displayed by customers when service delivery fails to meet their expectations[1]. This concept emphasizes the patient and forgiving attitude of customers in service contexts[2]. Tolerance behavior not only helps customers achieve inner balance, but also provides valuable opportunities for service providers to repair potential cracks in customer relationships caused by service deficiencies[3]. Especially in the tourism industry, tolerance behavior, as a manifestation of tourist citizenship behavior [4], is of great significance for promoting the long-term stable development of the tourism industry and enhancing competitive advantages [5, 6]. Therefore, the in-depth study of TTB by academia not only enriches customer behavior theory but also provides new perspectives and strategic ideas for enhancing tourism service quality.

As TTB has increasingly emerged as a focal point of academic inquiry, scholars have investigated the multiple factors influencing TTB from diverse perspectives. These factors can primarily be divided into two dimensions: organizational and individual factors. In terms of organizational factors, research encompasses critical elements such as rule-making[7] and hotel room pricing[8]. In terms of individual factors, it addresses customers' negative emotions[9] and their perceptions of climate change [10]. However, the above research context on TTB is mainly limited to the hotel industry[7-9], given that hotels represent relatively enclosed environments where guest interactions with staff constitute the primary service engagement. Conversely, tourist attractions are characterized by more open settings in which tourists engage with guides, area personnel, and local residents in varied ways. Consequently, findings related to tolerant behavior within hotel contexts cannot be directly extrapolated to scenarios involving tourist destinations [7-9]. Furthermore, interactions between tourists and local residents at these destinations are recognized as pivotal for fostering long-term sustainable development in tourism areas[11]. Previous studies have shown that interactive experiences within tourist attractions have a significant impact on tourists' behavior, such as stimulating their participation enthusiasm[12] and promoting word-of-mouth recommendations and the formation of sustained usage intentions[13]. Therefore, it is crucial to thoroughly investigate whether—and how—the interaction between tourists and local residents in open tourist attractions impacts TTB.

The references are as follows：

1. *Yi Y, Gong T. Customer value co-creation behavior: Scale development and validation. J Bus Res. 2013;66(9):1279-84.*
2. *Yang Y, Hu J. Self-diminishing effects of awe on consumer forgiveness in service encounters. Journal of Retailing and Consumer Services. 2021;60.*
3. *Hur J, Jang S. Is consumer forgiveness possible? International Journal of Contemporary Hospitality Management. 2019;31(4):1567-87.*
4. *Zhang H, Xu H. Impact of destination psychological ownership on residents’ “place citizenship behavior”. J Destin Mark Manage. 2019;14.*
5. *Torres-Moraga E, Rodriguez-Sanchez C, Sancho-Esper F. Understanding tourist citizenship behavior at the destination level. J Hosp Tour Manag. 2021;49:592-600.*
6. *Yao Y, Wang G, Ren L, Qiu H. Exploring tourist citizenship behavior in wellness tourism destinations: The role of recovery perception and psychological ownership. J Hosp Tour Manag. 2023;55:209-19.*
7. *Ma Shuang LX, Li Chunqing. Customer citizenship Behavior and misbehavior in the Context of Sharing economy: from the perspective of Social Dilemma Theory. Advances in psychological science. 2022;29(11):1920-35.*
8. *Alderighi M, Nava CR, Calabrese M, Christille J-M, Salvemini CB. Consumer perception of price fairness and dynamic pricing: Evidence from Booking.com. J Bus Res. 2022;145:769-83.*
9. *Xiong Wei HM, Huang Yuanfei. The elastic change of customer's negative emotion and tolerance in the context of hotel service failure tourism science. 2021;35:53-75.*
10. *Seekamp E, Jurjonas M, Bitsura-Meszaros K. Influences on coastal tourism demand and substitution behaviors from climate change impacts and hazard recovery responses. J Sustain Tour. 2019;27(5):629-48.*
11. *Eusebio C, Vieira AL, Lima S. Place attachment, host-tourist interactions, and residents' attitudes towards tourism development: the case of Boa Vista Island in Cape Verde. J Sustain Tour. 2018;26(6):890-909.*
12. *Wei MQ, Bai CH, Li CX, Wang HY. The effect of host-guest interaction in tourist co-creation in public services: evidence from Hangzhou. Asia Pac J Tour Res. 2020;25(4):457-72.*
13. *Moon H, Miao L, Hanks L, Line ND. Peer-to-peer interactions: Perspectives of Airbnb guests and hosts. International Journal of Hospitality Management. 2019;77:405-14.*

**Question 3：**The last paragraph of the introduction is not clear. The exposition of theoretical contributions is superficial. I suggest that the author reorganize the theoretical gaps and explain how they are filled.

**Response:** We sincerely appreciate your invaluable feedback regarding our introduction, particularly your observations concerning the theoretical contribution section. After a thorough review of your comments, we acknowledge that **the final paragraph of the introduction** lacks clarity and depth. Consequently, we have undertaken substantial revisions to this section. We revisited the existing literature and provided a comprehensive explanation of how our research addresses these gaps and delineates its specific contributions to the theoretical domain. Throughout the revision process, we ensured that our description of the theoretical gaps is both precise and exhaustive, while also clarifying and enhancing the feasibility of our proposed methods for addressing these gaps. Furthermore, we have reinforced the linkage between our theoretical contributions and subsequent research content to facilitate a clearer understanding for readers regarding our research objectives and significance. The revised section is as follows:

The literature contribution of this study to TTB is mainly reflected in the following aspects: Firstly, it underscores the critical role of HGI in shaping TTB within tourist destinations. By establishing HGI as a fundamental prerequisite for TTB, this research addresses whether tourists who engage more interactively are indeed more tolerant. Secondly, it elucidates the underlying mechanisms through which HGI influences TTB by incorporating PA and SWB. Lastly, this investigation is situated within the context of open tourist attractions, thereby broadening the scope of research on tolerance behavior in tourism and offering a theoretical framework for exploring analogous relationships in related fields. Consequently, this study paves new avenues for future inquiries into civic behavior among tourists. More broadly, it provides essential theoretical support and practical insights aimed at enhancing TTB in tourist destinations.

**Question 4：**Regarding the theoretical part, the SOR theory is a very familiar theory. I suggest removing the introduction of the SOR theory and placing the core views at the hypothesis derivation.

**Response:** We sincerely appreciate your invaluable suggestions concerning our approach to the SOR theory. Your insights are profoundly enlightening. We fully acknowledge your expectations for a more streamlined and focused theoretical framework, particularly in relation to the introduction of the SOR theory. Upon reviewing our content, we recognize that your recommendations hold significant merit. Therefore, we have decided to adopt your advice and reduce the introduction of the SOR theory, shifting more emphasis and space to the derivation of our hypotheses.

This adjustment aims to facilitate a clearer understanding of our research logic and core concepts for readers, thereby enhancing both coherence and readability throughout the paper.

**Question 5：**The derivation of the hypothesis is generally okay, but I suggest that the authors further refine it to make the derivation look more reasonable. Some expressions are not very professional, such as the subjective well-being theory on P6, I don't think it is a theory. Please check the derivation of the hypothesis again and update the necessary literature.

**Response:** We sincerely appreciate your meticulous review and invaluable suggestions regarding the deductive component of our hypothesis. We fully acknowledge the significance you have emphasized concerning the refinement and professional articulation of the deduction, as well as your corrections pertaining to the 'theory of subjective well-being' referenced on page 6. In light of your recommendations, we have resolved to enhance and refine our hypothesis deduction further, ensuring that each step is articulated with clarity, reasonableness, and logical coherence. Concerning the 'theory of subjective well-being' mentioned on page 6, we recognize that our expression may lack precision or professionalism; therefore, we will revisit this section to eliminate any inappropriate phrasing and incorporate relevant theoretical frameworks. Additionally, we will update necessary literature to guarantee that all references are current and authoritative in order to comprehensively enrich both the depth and breadth of this study. Below is the supplementary content:

## The mediating role of PA

PA is not a static phenomenon; rather, it is dynamic and evolves as tourists engage with their travel destinations, gradually fostering a robust emotional connection[14]. According to the SOR theory, individuals are significantly influenced by external stimuli and environmental factors, resulting in corresponding behavioral responses[35]. As a crucial component of travel experiences, HGI is anticipated to enhance the experiential value for tourists and further reinforce their PA towards the destination [36]. For instance, Woosnam discovered that interactions between tourists and local residents at World Heritage sites contribute to mutual attachment to these locations [37]. Additionally, Wang et al. highlighted that interactions between tourists and service providers positively influence PA levels[38]. Consequently, this study posits that HGI can effectively stimulate and strengthen tourists' attachment to their travel destinations. Based on this premise, we propose the following hypothesis:

**H2).** HGI has a significant positive effect on PA .

The emotional attachment individuals have to a place can significantly shape the values and behaviors of social groups[39], with personal identification and a sense of belonging serving as essential prerequisites for individual action. Previous studies have confirmed that PA has a positive and significant impact on tourists' environmental responsibility behaviors[40], intentions[41], and willingness to return[42]. In addition, combined with the attitude-behavior theory, PA, as a profound human-place emotional connection, is regarded as a key psychological factor that has an important impact on tourists' behavior and decision making[43]. Based on previous studies, this study posits that PA positively influences TTB. Consequently, the following hypothesis is proposed:

**H3).** PA has a significant positive effect on TTB.

The SOR theory posits that external environmental stimuli can elicit emotional responses within individuals, which subsequently lead to the development of corresponding attitudes and ultimately influence behavioral responses. PA is a kind of spiritual bond generated during the interaction between tourists and tourist destinations[14]. When individuals have a higher positive emotion towards the tourist destination, they will show a strong behavioral inclinations[43]. Moreover, PA serves as a mediating factor in various relational dynamics, such as those between restorative sensory perception and environmentally responsible behavior[40], as well as gastronomic experiences and behavioral intentions[41]. These findings underscore the viability of PA as a mediator and its substantial impact on individual behavior. Consequently, we hypothesize that PA may evoke specific behavioral responses when stimulated by HGI. Based on this premise, we propose the following hypotheses:

**H4).** PA plays a mediating role between HGI and TTB.

## The mediating role of SWB

Positive interaction is an important driver of tourists' SWB [48]. A lack of social interaction can lead to a diminished sense of well-being[49]. Conversely, even minimal social engagements can significantly enhance SWB in everyday life[50], contributing to greater happiness among individuals[51, 52].Existing literature indicates that teacher-student interactions positively and significantly influence students' classroom SWB[53],HGI on residents' happiness[54, 55], and human interaction on tourists' SWB[56]. Moreover, HGI offers tourists diverse travel experiences[12], which in turn can affect their SWB[57]. Therefore, the interaction between tourists and residents may have an impact on the SWB of tourists. Based on this premise, we propose the following hypothesis:

**H5).** HGI has a significant positive effect on SWB.

The theory of emotion regulation in psychology posits that an individual's emotional state and experiences can significantly influence their behavior[18]. For instance, as women's sense of happiness increases, they may exhibit greater resilience to the pressures associated with economic and caregiving responsibilities[58]. Likewise, the SWB of tourists during their travels has a positive and substantial effect on their decision-making behaviors regarding travel [19], intentions to revisit [59], and overall loyalty [60]. Drawing from prior research, this study asserts that SWB is a critical factor affecting tourists' behavioral intentions. Contented tourists are more inclined to demonstrate tolerance in their actions. Consequently, we propose the following hypothesis:

**H6).** SWB has a significant positive effect on TTB.

The SOR theory posits that external environmental stimuli can elicit emotional responses within the body, which subsequently influence behavioral reactions. Moreover, SWB serves as a mediator in various relational dynamics, such as the connection between landscape appeal and intentions for repeat visits[59], as well as the association between nostalgic emotions and intentions for repeat visits[61]. These findings underscore the viability of SWB as a mediating factor and its substantial impact on individual behavior. Consequently, we propose that SWB, representing an individual's most authentic feelings, can be conceptualized as an organism's subjective experience. In response to HGI stimulation, this subjective experience may provoke corresponding behavioral reactions. Building upon this premise, we further advance the following hypothesis:

**H7).** SWB plays a mediating role between HGI and TTB.

The references are as follows：

1. *Wei MQ, Bai CH, Li CX, Wang HY. The effect of host-guest interaction in tourist co-creation in public services: evidence from Hangzhou. Asia Pac J Tour Res. 2020;25(4):457-72.*
2. *Jin MLC, Y.Lee, C. K.Ahmad, M. S. Effects of Place Attachment and Image on Revisit Intention in an Ecotourism Destination: Using an Extended Model of Goal-Directed Behavior. Sustainability-Basel. 2020;12(18).*
3. *Xu J, Xie B, Chung B. Bridging the Gap between Affective Well-Being and Organizational Citizenship Behavior: The Role of Work Engagement and Collectivist Orientation. Int J Environ Res Public Health. 2019;16(22).*
4. *Skard SK, E. S., Sjastad HT, H. How virtual reality influences travel intentions: The role of mental imagery and happiness forecasting. Tourism Manage. 2021;87.*
5. *Darley JM, & Gilbert, D. T. Social psychological aspects of environmental psychology. Handbook of social psychology. 1985;2:949–92.*
6. *Bi J, Wang B, Lu F. Does Host-Guest Interaction Stimulate Tourists’ Citizenship Behavior? A Combination of Social Exchange Theory and Cognitive Appraisal Theory. Forests. 2024;15(7).*
7. *Woosnam KM, Aleshinloye KD, Ribeiro MA, Stylidis D, Jiang J, Erul E. Social determinants of place attachment at a World Heritage Site. Tourism Manage. 2018;67:139-46.*
8. *Wang S, Lehto X, Cai LP, Behnke C, Kirillova K. Travelers' Psychological Comfort with Local Food Experiences and Place Attachment. J Hosp Tour Res. 2021.*
9. *D C. The facets of place. Springer: Boston,MA; 1997.*
10. *Wu K, Guo Y, Han X. The relationship research between restorative perception, local attachment and environmental responsible behavior of urban park recreationists. Heliyon. 2024;10(15).*
11. *Akoğul E, and Gökalp N. Selçuk. Effect of international food tourists’ food experiences on place attachment and behavioral intentions: The moderating role of food variety seeking tendency Journal of Vacation Marketing 2024:13567667241230345.*
12. *Pu B, Wei J, Zhou L, Li L. A study on the impact of tourism destination image and local attachment on the revisit intention: The moderating effect of perceived risk. Plos One. 2024;19(1).*
13. *Isa SM, Ariyanto HH, Kiumarsi S. The effect of place attachment on visitors' revisit intentions: evidence from Batam. Tourism Geogr. 2020;22(1):51-82.*
14. *Kim K, Uysal M, Sirgy MJ. How does tourism in a community impact the quality of life of community residents? Tourism Manage. 2013;36:527-40.*
15. *Woo E, Kim H, Uysal M. Life satisfaction and support for tourism development. Ann Tourism Res. 2015;50:84-97.*
16. *Johnson DCSaDM. Avowed happiness as an overall assessment of the quality of life. Social Indicators Research. 1978;5(4):475-92.*
17. *Durkin J, Joseph S. Growth Following Adversity and Its Relation with Subjective Well-Being and Psychological Well-Being. Journal of Loss and Trauma. 2009;14(3):228-34.*
18. *Su Lujun, He Xuehuan, Hu Dongbin. Research Progress and Implications of Tourists' Subjective Well-being [J]. Journal of Sichuan Normal University (Social Sciences Edition), 2019, 46(02): 83-92.*
19. *Baumeister RF, and Mark R. Leary. The need to belong desire for interpersonal attachments as a fundamental human motivation. Psychological bulletin. 1995;117(3):497.*
20. *Gunaydin G, Oztekin H, Karabulut DH, Salman-Engin S. Minimal Social Interactions with Strangers Predict Greater Subjective Well-Being. J Happiness Stud. 2021;22(4):1839-53.*
21. *Sandstrom GM, Dunn EW. Social Interactions and Well-Being: The Surprising Power of Weak Ties. Pers Soc Psychol Bull. 2014;40(7):910-22.*
22. *Kroencke L, Harari GM, Back MD, Wagner J. Well-being in social interactions: Examining personality-situation dynamics in face-to-face and computer-mediated communication. Journal of Personality and Social Psychology. 2023;124(2):437.*
23. *Xiao M, Tian Z, Xu W. Impact of teacher-student interaction on students’ classroom well-being under online education environment. Education and Information Technologies. 2023;28(11):14669-91.*
24. *Chen Y, Cottam E, Lin Z. The effect of resident-tourist value co-creation on residents’ well-being. J Hosp Tour Manag. 2020;44:30-7.*
25. *Wang H, Sha H, Liu L, Zhao H. Exploring the Relationship between Perceived Community Support and Psychological Well-Being of Tourist Destinations Residents. Int J Env Res Pub He. 2022;19(21).*
26. *van Esch P, Cui Y, Das G, Jain SP, Wirtz J. Tourists and AI: A political ideology perspective. Ann Tourism Res. 2022;97.*
27. *Yang S, Liu Y, Xu L. The effect of food tourism experiences on tourists' subjective well-being. Heliyon. 2024;10(3).*
28. *Ranganathan M, Stern E, Knight L, Muvhango L, Molebatsi M, Polzer-Ngwato T, et al. Women's economic status, male authority patterns and intimate partner violence: a qualitative study in rural North West Province, South Africa. Cult Health Sex. 2022;24(5):717-34.*
29. *Dat LT, Wu HC, Li TN, Huang WS, Liou GB, Hsieh CM. The effects of landscape fascination on subjective well‐being and revisit intention: Evidence from agritourism destinations. International Journal of Tourism Research. 2024;26(1).*
30. *Liu SP, Li SY, Chen YQ, Zheng TX. Examining relationships among food's perceived value, well-being, and tourists' loyalty. J Vacat Mark. 2023;29(2):161-74.*
31. *Karagöz D, and Haywantee Ramkissoon. Nostalgic emotions, meaning in life, subjective well-being and revisit intentions. Tourism Management Perspectives. 2023;48:101159.*

**Question 6：**The analysis process of the two empirical studies is generally reasonable, but there are two major problems for the author to consider: First, neither of the two questionnaire studies has carried out the necessary common method bias analysis, and second, when considering the relationship between variables, the interference of irrelevant variables is not considered.

**Response:** We sincerely appreciate your meticulous review and insightful comments regarding our empirical research analysis process. We fully recognize and concur with the two significant issues you highlighted, as well as their critical importance to the overall quality of our study. Concerning the first point you raised, we acknowledge that neither of the two questionnaire studies conducted a necessary analysis for common method bias; this represents an oversight in our study design. Common method bias is indeed a vital consideration, particularly in research relying on self-reported data. Consequently, we have resolved to enhance this aspect in **Study 1** and **Study 2** by employing appropriate methodologies (such as the Harman single-factor test) to evaluate and mitigate any potential common method bias. The supplementary content is detailed below:

Study 1

To mitigate the influence of common method bias, this study implemented pre-test control measures, including randomizing the order of items, and employed the Harman single-factor method to assess the presence of common method bias within the data. The findings indicated that measurement items were consolidated into four distinct factors. Notably, the first factor accounted for only 22.612% of the total variance across all items, falling below the critical threshold of 40%, thereby suggesting that effective controls were in place to address potential common method variance in this study.

Study 2

To mitigate the influence of common method bias, this study implemented pre-test control measures, including randomizing the order of items, and employed the Harman single-factor method to assess the presence of common method bias within the data. The findings indicated that the measurement items were consolidated into four distinct factors. Notably, the first factor accounted for only 24.125% of the total variance among all items, which falls below the critical threshold of 40%, thereby suggesting that effective controls were in place to address potential common method variance in this study.

Concerning the second point you raised regarding the exclusion of irrelevant variables in examining the relationships between variables, we acknowledge that this is a matter warranting careful consideration. **Firstly,** our study primarily aims to investigate the fundamental relationship between independent and dependent variables. To present and elucidate this critical pathway and its effects more clearly, we opted to simplify the model by omitting discussions on control variables. **Secondly,** our decision to forgo an in-depth analysis of control variables stems from concerns about their potential interference and misleading implications. This approach enhances the report's conciseness, enabling readers to better grasp the focus and core findings of our research. **Lastly,** we reviewed numerous articles employing AMOS software for structural equation modeling; notably, none incorporated control variables but instead directly analyzed their models. For instance, **Yin** examined the relationship between cultural confidence and community civic behavior while **Su** investigated how tourist service quality relates to subjective well-being. Our objective is to underscore the primary contributions of this study while ensuring that readers have a clear understanding of our main hypotheses and conclusions. Consequently, we did not consider irrelevant variable interference when analyzing variable relationships.

The references are as follows：

1. *Yin, Jie, Yingchao Ji, and Yensen Ni. "How does cultural confidence awaken community citizenship behaviors? The moderating effect of cultural involvement." Journal of Hospitality and Tourism Management.2023; 55: 425-434.*
2. *He, Xuehuan, Lujun Su, and Scott R. Swanson. "The service quality to subjective well-being of Chinese tourists connection: A model with replications." Current Issues in Tourism.2020; 23(16): 2076-2092.*

**Question 7：**Regarding why these two places were chosen as case locations, I think the author should have an explanation. In addition, I think that these two studies can actually be merged into one study. You can merge them to try to draw universal conclusions, or you can use MGA to do the multi-group analysis effect comparison. Currently, study 2 is completely repeating study 1, which looks very redundant.

**Response:** Thank you very much for your insightful review and valuable comments regarding our study. Concerning the selection of the two case locations you mentioned, we acknowledge the necessity of providing a clear rationale in the paper to enhance both its validity and persuasiveness. We apologize for this oversight and will incorporate relevant explanations in our forthcoming revision. With respect to your suggestion of merging the two studies into one and conducting multi-group analyses to derive overarching conclusions, we have given it careful consideration.We recognize that multi-group analyses can elucidate differences and trends among various groups, which is crucial for comprehensive research. However, in our current study, our primary objective is to validate the robustness of our findings by collecting and analyzing data from two distinct types of tourist destinations. Given that no significant differences were identified between these two case locations in our results, we are concerned that introducing multi-group analyses may unnecessarily lengthen the article and potentially lead to overinterpretation or mislead readers. Furthermore, through literature review, we have observed that numerous scholars have examined multiple studies separately to substantiate their conclusions; for instance, **Wu et al.** investigated the impact of destination psychological ownership on residents' "place citizenship behavior" through surveys conducted among residents in Zhuhai and Hangzhou; similarly, **Su et al.** explored the relationship between visitor service quality and subjective well-being via surveys administered to tourists in Xiamen and Yuelu Mountain. Therefore, we opted not to include a discussion on conducting multi-group analyses for these two case locations within this study.

The references are as follows：

1. *Wu, M. Y., Wu, X., Li, Q. C., & Tong, Y. . Community citizenship behavior in rural tourism destinations: Scale development and validation. Tourism Management.2022;89,104457.*
2. *He, Xuehuan, Lujun Su, and Scott R. Swanson. "The service quality to subjective well-being of Chinese tourists connection: A model with replications." Current Issues in Tourism.2020, 23(16): 2076-2092.*

**Question 8：**The part of theoretical contribution needs to be further deepened, and it seems that the research value of this paper is not highlighted. It is suggested that the author have a deeper dialogue with the existing literature, inform the readers what the gap is, and how your research fills it.

**Response:** We sincerely appreciate your invaluable feedback regarding our theoretical contribution. We fully acknowledge the points you have raised and recognize that the research value of this article may not have been adequately emphasized in the manuscript. Your suggestions have guided us toward improvement, specifically by engaging in a more thorough dialogue with existing literature, clearly delineating the research gaps, and articulating how our study addresses those gaps. To accomplish this, we have undertaken a comprehensive literature review in the **first two sections** of our **theoretical contribution**, explicitly identifying the limitations of current research and clearly articulating how our work fills these voids. These modifications aim to provide a more robust presentation of the significance and innovation inherent in this study. The specific changes are as follows:

Firstly, this study significantly enhances the existing literature on tourist citizenship behaviors in tourist destinations by establishing that HGI serves as a critical prerequisite for TTB. While HGI demonstrably influences various aspects of tourists' conduct, it remains uncertain whether increased interactions with local residents correlate with a higher propensity for exhibiting tolerance behavior. Prior research has predominantly focused on the effects of HGI on other tourist behaviors, such as participation behavior[12] eco-friendly practices[25, 26], and sustained usage intention[13]. Nevertheless, this study elucidates the pivotal role of HGI in fostering TTB by demonstrating that tourists’ engagement with residents can inspire them to cultivate an intention toward tolerance, thereby contributing to the broader discourse on tolerance behavior.

Secondly, this study substantiates the chain mediation role of PA and SWB in the relationship between HGI and TTB, thereby elucidating the underlying mechanisms through which HGI fosters TTB. Prior research has emphasized the necessity for a comprehensive investigation into the formation mechanisms of TTB within tourist destinations, particularly concerning various influencing factors[7-10]. This study employs attitude-behavior theory to examine tolerance behavior while validating the mediating roles of PA and SWB, thus enhancing our understanding of TTB in the context of tourist destinations[36, 57]. Moreover, previous scholars have indicated that further empirical investigations are essential to explore the determinants of PA and SWB among tourists at these locations. Our findings reveal that HGI serves as a significant catalyst for both PA and SWB, contributing novel insights to existing literature on tourist destinations.

The references are as follows：

1. *Ma Shuang LX, Li Chunqing. Customer citizenship Behavior and misbehavior in the Context of Sharing economy: from the perspective of Social Dilemma Theory. Advances in psychological science. 2022;29(11):1920-35.*
2. *Alderighi M, Nava CR, Calabrese M, Christille J-M, Salvemini CB. Consumer perception of price fairness and dynamic pricing: Evidence from Booking.com. J Bus Res. 2022;145:769-83.*
3. *Xiong Wei HM, Huang Yuanfei. The elastic change of customer's negative emotion and tolerance in the context of hotel service failure tourism science. 2021;35:53-75.*
4. *Seekamp E, Jurjonas M, Bitsura-Meszaros K. Influences on coastal tourism demand and substitution behaviors from climate change impacts and hazard recovery responses. J Sustain Tour. 2019;27(5):629-48.*
5. *Wei MQ, Bai CH, Li CX, Wang HY. The effect of host-guest interaction in tourist co-creation in public services: evidence from Hangzhou. Asia Pac J Tour Res. 2020;25(4):457-72.*
6. *Moon H, Miao L, Hanks L, Line ND. Peer-to-peer interactions: Perspectives of Airbnb guests and hosts. International Journal of Hospitality Management. 2019;77:405-14.*
7. *Wang WF, Wu JX, Wu MY, Pearce PL. Shaping tourists' green behavior: The hosts' efforts at rural Chinese B&Bs. J Destin Mark Manage. 2018;9:194-203.*
8. *Li SH, Liu M, Wei M. Host sincerity and tourist environmentally responsible behavior: The mediating role of tourists' emotional solidarity with hosts. J Destin Mark Manage. 2021;19.*
9. *Bi J, Wang B, Lu F. Does Host-Guest Interaction Stimulate Tourists’ Citizenship Behavior? A Combination of Social Exchange Theory and Cognitive Appraisal Theory. Forests. 2024;15(7).*
10. *Yang S, Liu Y, Xu L. The effect of food tourism experiences on tourists' subjective well-being. Heliyon. 2024;10(3).*

To enhance your experience, we have highlighted the modified sections in blue within the text. We sincerely appreciate your invaluable feedback and support. Should you identify any areas for improvement, we would be eager to receive your suggestions and will strive diligently to implement further enhancements. Thank you once again for your dedicated efforts and professional guidance.
