# Supplementary material for: Does host-guest interaction promote tolerance behavior? The mediating role of place attachment and subjective well-being
Source: PLoS One. 2025 May 28;20(5):e0323355. doi: 10.1371/journal.pone.0323355 (PMC12118835; doi:10.1371/journal.pone.0323355)
Supplement: Attachment — Submitted filename: Response_to_Reviewers_auresp_2.docx [file pone.0323355.s003.docx]

**Response Letter**

**Dear Reviewers:**

Firstly, I would like to extend my sincere gratitude to you for the invaluable feedback and suggestions provided on my previous submission. Your comments have been instrumental not only in advancing the improvement of my research but also in significantly enhancing the overall quality of the manuscript.
 After a meticulous revision process, I have thoroughly addressed each suggestion put forth by the reviewers. I am confident that these enhancements have substantially improved the content, logic, and clarity of the manuscript, bringing it into closer alignment with your publication standards.
 If there are any additional areas for refinement, I would greatly appreciate your insights and will endeavor to further enhance the manuscript. Once again, I express my profound appreciation for your dedicated efforts and professional guidance.

I look forward to your positive response.

Yours sincerely,

Ke Wu

**Reviewer: 1**

We wish to convey our sincere gratitude to the reviewers for dedicating their valuable time, despite their busy schedules, to evaluate our manuscript. The constructive suggestions provided by the reviewer have significantly enhanced the quality of our work. We extend our heartfelt appreciation for your efforts.

In response to the points you have raised, we will address each one individually as follows:

**Question 1：**First, the introduction lacks logical coherence in presenting the theoretical background and research context. It is recommended to reorganize the introduction, starting with a clear overview of the theoretical background of tolerance behavior, followed by the impact of host-guest interaction on tolerance behavior, and finally, leading to the research question and objectives.

**Response:** We wish to convey our deepest appreciation to the esteemed expert for the invaluable feedback provided on our **introduction section**. The initial draft suffered from a lack of logical coherence when presenting the theoretical and research background. In response to the expert's recommendations, we have thoroughly restructured the **introduction**. Firstly, we now present a concise yet comprehensive overview of the theoretical underpinnings of tolerance behavior, encompassing its definition, significance, and the current state of research, thereby offering readers a thorough and nuanced understanding. Secondly, we delve into the influence of host-guest interactions on tolerance behavior, examining the inherent connections and underlying mechanisms, which further solidify and deepen the theoretical framework of our paper. Lastly, we articulate the research problem and objectives, delineating the research direction and focal points of the study, thus providing a clear and structured research framework for our audience. Once again, we extend our heartfelt gratitude for the meticulous guidance and insightful suggestions offered by the expert.

The modifications are outlined as follows:

In recent years, the swift advancement of an open economy and the dissemination of civilized tourism principles have prompted a growing number of tourists to willingly engage in pro-social behaviors that benefit tourism enterprises or destinations, referred to as tourist civic behaviors (1). These behaviors are characterized by their spontaneous and voluntary nature, generating value for stakeholders without being obligatory(2, 3). They encompass activities such as providing positive word-of-mouth recommendations (4), offering assistance, giving feedback(5), and demonstrating tolerance(2). Within the value co-creation framework between tourists and service providers (2), tourists' tolerance behavior assumes a pivotal role in fostering the long-term sustainable development of the tourism sector and enhancing its competitive edge (5, 6). Consequently, tolerance behavior has garnered increasing scholarly attention(2), as it not only aids in balancing the often uneven relationship between customers and service providers(7), but also actively contributes to the enhancement of tourists' travel experiences and overall tourism quality.

However, existing research on tourist tolerance behavior (TTB) predominantly focuses on the hospitality sector(8, 9, 10, 11). It is important to highlight that, within the relatively confined environment of hotels, customer interactions primarily occur between guests and hotel personnel. Conversely, tourist destinations present a more open setting where visitors engage not only with destination staff, such as tour guides and park attendants, but also regularly with local inhabitants through unstructured encounters. Consequently, the direct application of tolerance behavior influence mechanisms from the hotel context to tourist destination scenarios is constrained by these differences (8, 9, 10).

Next, we delve into the relationship between tourists and tourist destinations, with particular emphasis on the host-guest interaction (HGI), which is widely recognized as a pivotal factor in the sustainable development of tourist destinations(12). The HGI, as a distinctive tourism attraction element, significantly influences tourists' perceptions, attitudes, and behaviors (13). In tourism resources of universal value and substantial significance, such as natural and cultural heritage sites, tourists are inclined to place greater importance on the quality of host-guest interaction (14). Studies indicate that when tourists form a "warm partnership" with local residents, this relationship effectively enhances their engagement(15) and fosters the creation of positive word-of-mouth and sustained visitation intentions(4). Consequently, in both open natural heritage and cultural heritage tourist destinations, thoroughly investigating how HGI impacts TTB not only contributes to the enrichment of customer behavior theory but also offers novel perspectives and strategies for enhancing tourism service quality.

**Question 2:** Second, as an empirical study, I find the logical connection between Study 1 and Study 2 quite weak. Even though the authors have explained the purpose of conducting both studies, it does not adequately support the claim of generalizability for the model. Additionally, upon reviewing the latter part of the manuscript, it appears that much of the empirical content is focused on data description without adequately explaining why. This may be related to insufficient theoretical elaboration in the earlier sections, which also results in a superficial discussion in the theoretical analysis section. I recommend further referencing standardized multi-study research designs and paradigms.

**Response:** We wish to convey our deepest appreciation to the esteemed expert for the meticulous review of our research and the invaluable feedback provided. Concerning the weak logical connection between **Study 1 and Study 2** that you have highlighted, we hold this matter in high regard and have implemented appropriate measures.

First, in this paper, **Study 1** is designed to examine whether interpersonal interactions at natural heritage open-access sites influence tourists' tolerance behavior. Meanwhile, **Study 2** seeks to validate the robustness of **Study 1**’s findings by replicating the investigation across various types of cultural heritage open-access sites, thereby enhancing the external validity of the research.

Additionally, the approach of conducting studies in diverse case sites to confirm the robustness of conclusions has been adopted by prior researchers. **For instance, Su et al. executed three surveys to explore the connection between visitor service quality and subjective well-being, as well as to assess the consistency of the proposed model across different destinations.**

*He, Xuehuan, Lujun Su, and Scott R. Swanson. "The service quality to subjective well-being of Chinese tourists connection: A model with replications." Current Issues in Tourism.2020, 23(16): 2076-2092.*

Furthermore, we have refined and adjusted the descriptions of **Study 1 and Study 2** to ensure the overall coherence and persuasiveness of the research.

The modifications are outlined as follows:

***Study Site* section:** Finally, gathering samples from two distinct categories of tourist attractions and performing independent data analyses contributes to enhancing the validity and robustness of our research outcomes.

***Measurement model* part of Study 1:** By analysing the data with Amos software, the measurement model of study 1 was well fitted (see Table 3).Cronbach's alpha values ranged from 0.830 to 0.938, all greater than 0.7, meeting the criteria(73). Factor loadings for all items (see Table 4) were greater than 0.5, which is statistically significant (p < 0.001) and meets the criterion(73). The composite reliability (CR) values ranged from 0.827 to 0.938, with each variable's CR exceeding the minimum threshold of 0.7 as recommended by Hair et al.(74). Additionally, the average variance extracted (AVE) for all constructs ranged from 0.549 to 0.791, satisfying the established criteria(73). Therefore, in terms of the overall data, the samples in Study 1 all have high reliability, validity and internal consistency.

***Data collection* part of Study 2:** To examine the proposed model’s stability across destinations.

***Measurement model* part of Study 2****:** The measurement model of Study 2 was well fitted (see Table 3), Cronbach's alpha values ranging from 0.864 to 0.924, all exceeding the threshold of 0.7, thus satisfying the reliability criteria. Factor loadings for all items, as presented in Table 6, were greater than 0.5 and achieved statistical significance (p < 0.001). The composite reliability (CR) of each variable ranged from 0.866 to 0.918, surpassing the 0.7 benchmark, while the average variance extracted (AVE) for all constructs ranged from 0.618 to 0.807, well above the minimum requirement of 0.5(73). Therefore, from perspective of the overall data, the samples in Study 2 all have high reliability, validity and internal consistency.

Concerning the critique that the empirical content was overly focused on data description and lacked in-depth analysis, we have undertaken a thorough revision of the **discussion section**. By integrating real-world case studies and theoretical frameworks, we now offer a more profound and nuanced interpretation and examination of our research findings, aiming to provide readers with richer and more insightful analytical perspectives.

The modifications are outlined as follows:

## Discussion

This study selected tourists from two distinct tourist destinations as research subjects to explore the intrinsic relationships between HGI, PA, SWB, and TTB through the construction of a conceptual model. By systematically collecting and analyzing feedback from tourists at these destinations, this study has not only enriched the body of knowledge on tolerance behavior but also expanded the existing research scope.

Firstly, we have confirmed that HGI exerts a significant positive influence on TTB. The HGI is considered a key driver in promoting the long-term sustainable development of tourist destinations**(12)**. Prior research has predominantly examined HGI as a prerequisite or moderating variable influencing residents' behaviors **(24)**, with a focus on its effects on tourists' green behaviors**(21)** and environmentally responsible actions **(22)**. Nevertheless, the mechanisms through which HGI impacts TTB are more intricate and multifaceted, and existing research remains inadequate. Tolerant behavior not only facilitates tourists' internal harmony but also offers valuable opportunities for service providers to rectify damaged customer relationships resulting from service failures **(76)**.

Secondly, our research indicates that PA serves as a mediating factor between HGI TTB. PA signifies the evolving emotional connection between tourists and tourist destinations **(31)**, while HGI enhances this attachment. This finding aligns with prior studies demonstrating that HGI significantly influence tourists' cognitive and emotional responses**(33)**. Furthermore, the strengthening of the human-place emotional bond positively impacts TTB, corroborating existing research that tourists' emotional attachment and sense of identity shape their attitudes toward tourist destinations, thereby influencing their behaviors and decisions**(39)**. We propose that in the context of open tourist destinations, where tourists have easy access to communication with local residents, this interaction facilitates the reinforcement of the emotional bond between tourists and destinations, leading to greater willingness to tolerate imperfections or negative aspects of the tourist experience.

Thirdly, we have confirmed that SWB acts as a partial mediator between HGI and TTB. SWB represents individuals' cognitive assessments of their own life satisfaction **(42)**. Prior research predominantly concentrated on the influence of HGI on residents' well-being**(50, 51)**, whereas this study broadens the scope to include tourists at tourism destinations, offering a novel perspective on the positive effects of HGI on tourists. Our findings suggest that within the tourism context, HGI can enhance tourists' SWB, and when tourists experience elevated levels of well-being at their destinations, they are more inclined to exhibit understanding and tolerance. This aligns with the theory of emotion regulation, which posits that an individual's emotional state substantially impacts their behavior **(54)**. Specifically, the communication and interaction between tourists and local residents at tourism destinations not only influence the quality of tourists' travel experiences but also profoundly affect their SWB. Tourists with higher SWB levels are more likely to adopt a positive attitude towards tourism-related unfavorable situations.

To address the issue of insufficient theoretical elaboration, which has led to a superficial theoretical analysis, we have implemented corresponding enhancements. In the **theoretical analysis section**, we have expanded the depth of our analysis of research findings and the extraction of theoretical contributions, with the aim of solidifying and deepening the theoretical foundation of the study. Additionally, we have proactively adopted standardized multi-research designs and paradigms to ensure the full scientific rigor and robustness of the study.

The modifications are outlined as follows:

## Theoretical implications

This study makes several contributions. Firstly, by investigating the relationships among tourists' HGI, PA, SWB, and TTB, it not only broadens the scope of TTB research and its antecedents but also provides valuable insights for destination managers aiming to enhance destination development. While previous literature on tolerance behavior has predominantly centered on hotel environments(8, 9, 10), it has not extensively explored the causal relationship between open-space tourists' HGI and TTB through multiple case studies. It is important to note that open-space tourism destinations differ significantly from closed-space hotels, where customer expectations are primarily focused on highly personalized services, and their tolerance is mainly directed towards minor service errors or shortcomings. In contrast, open-space tourists seek to experience natural beauty, cultural heritage, or entertainment activities, and their tolerance is more often related to inconveniences encountered during their tourism experiences. Considering the positive influence of tourists on destination development (5, 6), this study innovatively extends the application of TTB research by validating the impact of open-space tourists' HGI on TTB.

Secondly, this study selected two distinct types of tourist destinations for data collection: the Two Rivers and Four Lakes, which are natural heritage sites, and Xingping Ancient Town, a cultural heritage site. This selection not only encompasses the diversity of tourist destinations but also offers a valuable empirical foundation for investigating the similarities and differences in visitor behavior patterns across different types of tourist destinations. The data analysis from these two case study sites underscores the broad applicability and robustness of the research findings. It enhances the credibility of the study and provides a solid foundation for future research to promote and apply relevant theories in various types of tourist destinations.

Finally, prior research has emphasized the necessity of a comprehensive investigation into the formation mechanisms of (TTB in tourist destinations, particularly focusing on the examination of diverse influencing factors (8, 9, 10, 11). This study adopts the Attitude-Behavior Theory and applies it to the analysis of tolerance behaviors, thereby validating the sequential transmission from PA to SWB, which enhances our understanding of TTB in tourist settings. Furthermore, earlier scholars have recommended conducting additional empirical research to delve into the determinants of PA and SWB among tourists in tourist destinations(14, 53). Our findings indicate that HGI serves as a significant catalyst for PA and SWB, contributing novel insights to the existing body of knowledge on tourist destinations.

We would like to extend our sincere gratitude once more for your invaluable advice and suggestions.

**Question 3:**Third, I have concerns about the measurement of TTB. After reviewing the cited literature, I found that there is no direct relationship between the cited scales and TTB, which raises questions about the validity of the measurement.

**Response:** We are deeply grateful for your comprehensive review and meticulous attention to the TTB measurement method. We fully comprehend your concerns regarding the scale's validity and wish to provide a detailed explanation of our considerations and validation procedures to address these concerns.

In academic research, the appropriateness of measurement scales is indeed an issue that demands meticulous attention, particularly in cross-cultural settings. As highlighted by **Professor** **Chen Xiaoping** in Chapter 7 of "Research Methods in Organization and Management Studies (Third Edition)," the traditional cultural context of China markedly diverges from that of the West, and these distinctions can substantially influence the development and application of scales. Consequently, it is imperative to exercise particular caution in the selection and utilization of scales.

**Xie et al. (2006)** selectively utilized specific items from the scale to assess personality factors associated with self-evaluation. Likewise, **Zhang et al. (2019)** effectively modified the scale originally developed by **Van Dyne and Pierce (2004)** for studying organizational citizenship behavior, adapting it to suit the context of tourist destinations. Drawing on these precedents, our study has refined the items proposed by **Yi et al. (2013)** to evaluate tolerance behavior among customers, ensuring that they are more applicable to the specific scenarios encountered at tourist destinations, thereby facilitating a more accurate portrayal of tourists' tolerance behavior.

To further ensure the validity and appropriateness of the adapted scale, we invited three seasoned tourism experts to critically evaluate the scale. Their input provided invaluable suggestions regarding the phrasing and structure of the questionnaire.

We undertook preliminary research and gathered a substantial amount of data. Subsequently, through the analysis of this data, we performed an exploratory factor analysis to validate the reliability and appropriateness of the Tolerance Behavior Scale.

The analysis results indicate that the scale exhibits satisfactory reliability and validity, enabling consistent measurement of tourist tolerance behavior. This outcome further substantiates the rationale behind our scale revision.

1. *Xie, Jia Lin, Jean‐Paul Roy, and Ziguang Chen. "Cultural and individual differences in self‐rating behavior: an extension and refinement of the cultural relativity hypothesis." Journal of Organizational Behavior: The International Journal of Industrial, Occupational and Organizational Psychology and Behavior 27.3 (2006): 341-364.*
2. *Zhang, Hui, and Honggang Xu. "Impact of destination psychological ownership on residents’“place citizenship behavior”." Journal of Destination Marketing & Management 14 (2019): 100391.*
3. *Van Dyne L, Pierce J L. Psychological ownership and feelings of possession: Three field studies predicting employee attitudes and organizational citizenship behavior[J]. Journal of Organizational Behavior: The International Journal of Industrial, Occupational and Organizational Psychology and Behavior, 2004, 25(4): 439-459.*
4. *Yi Y, Gong T. Customer value co-creation behavior: Scale development and validation[J]. Journal of Business research, 2013, 66(9): 1279-1284.*

We trust that our explanation has mitigated your concerns, and we eagerly anticipate your valuable feedback and recommendations for our research.

**Question4：**Lastly, the overall writing quality, both in terms of logic and style, is relatively rough and needs significant improvement.

**Response:** We are deeply grateful for your meticulous review and insightful comments regarding the writing quality of the article. We have fully acknowledged the deficiencies in the logical structure and stylistic presentation of the article, and have initiated a comprehensive revision process. In response to your recommendations, we have meticulously revised and refined the entire document, with a focus not only on sentence construction and grammatical accuracy but also on enhancing the logical coherence and clarity of expression. It is our sincere hope that these enhancements will render the article more comprehensible and accessible, thereby more effectively communicating our research findings and conclusions.

For your convenience, we have highlighted the revised sections in blue within the document. We are deeply grateful for your valuable feedback. We remain committed to enhancing the quality of our research. Should there be any additional areas for improvement, we would be most appreciative of your further recommendations and will endeavor to make the necessary enhancements. Once more, we thank you for your diligent efforts and expert guidance.

**Reviewer: 3**

We wish to extend our sincere appreciation for your constructive feedback and insightful recommendations regarding our work. As detailed below, all comments have been meticulously evaluated and effectively incorporated into the revised manuscript. We are deeply grateful for your support.

In response to all the points you have raised, we will address each one in turn:

**Question 1:** It would be very helpful if the authors can provide more information about the research area. What is “Two Rivers and Four Lakes” and what is “Xingping Ancient Town”? Please provide a background introduction for non-local readers.

**Response:** We are deeply grateful for your meticulous review and insightful feedback. Based on your recommendations, we have provided comprehensive enhancements to the research background. The revised content is as follows:

## Study Site

Guilin, renowned as one of China’s premier tourist destinations, holds a distinguished place among the first batch of National Famous Historical and Cultural Cities. It is celebrated for its unparalleled natural beauty, often lauded with the phrase “Guilin’s landscape is the finest under heaven.” A notable highlight is the Two Rivers and Four Lakes Scenic Area, a 5A-rated attraction that falls under the category of natural heritage sites. This scenic belt encircles the city center of Guilin, presenting a captivating ring-shaped landscape that integrates the Li River, Peach Blossom River, Banyan Lake, Fir Lake, Laurel Lake, and Mulong Lake into a harmonious urban tableau. Another noteworthy site is the Xingping Ancient Town Scenic Area, a 4A-rated attraction categorized as a cultural heritage site. Known as “the luminous pearl on the Li River,” Xingping Ancient Town is situated in Yangshuo County, Guilin City, and boasts over a millennium of history. The town is replete with Ming and Qing Dynasty architecture and invaluable cultural artifacts, offering visitors an opportunity to delve into its rich historical and cultural tapestry.

We are grateful for your valuable feedback and hope that it will assist readers in gaining a deeper understanding of our research.

**Question 2：**Some recent research related to roles of SWB and PA would provide helpful insights to develop the theory and hypotheses. For example, “Community resilience in city emergency: Exploring the roles of environmental perception, social justice and community attachment in subjective well-being of vulnerable residents” provides a new theory combining PA (where they recognized as CA) and SWB to community development.

**Response:** We are deeply grateful for your comprehensive review and insightful recommendations. After a meticulous examination of the literature you cited, we have refined and expanded our initial hypothesis concerning the influence of PA on SWB. We have substituted outdated references with more current research on the correlation between PA and SWB, incorporating the studies you highlighted, **specifically references 61, 62, and 63. Notably, we have revised reference 68 to align with the article you suggested**.

The following details outline the specific enhancements made:

Research demonstrates a strong positive correlation between PA and SWB(61). PA benefits individuals both physically and psychologically, thereby potentially enhancing SWB(62) and influencing individual behavioral responses(63). Neighborhood well-being is affected by PA(64). PA facilitates the development of individual SWB(65). On the basis of consistent PA, SWB can be further promoted(66). Additionally, PA acts as a mediator in the relationship between local social identification and well-being(67), while community attachment mediates the relationship between perceptions of the community environment and residents' SWB(68).

61. *Wu D, Shen C, Wang E, et al. Impact of the perceived authenticity of heritage sites on subjective well-being: A study of the mediating role of place attachment and satisfaction[J]. Sustainability, 2019, 11(21): 6148.*
62. *Chen J J, Liu L F, Chen S M. The Role of Place Attachment in the Relationship between Attitudes toward Aging and Subjective Well-Being among Community-Dwelling Older Adults in Taiwan[C]//Healthcare. MDPI, 2024, 12(10): 981.*
63. *Li J, Liu C, Yuan J J, et al. Understanding Destination Immersion in Rural Tourism: The Effects of Destination Fascination and Resident–Tourist Interaction[J]. Journal of Travel Research, 2024: 00472875241257269.*
68. *Wang Q C, Liu X, Jian I Y, et al. Community resilience in city emergency: Exploring the roles of environmental perception, social justice and community attachment in subjective well-being of vulnerable residents[J]. Sustainable Cities and Society, 2023, 97: 104745.*

We are grateful for your invaluable guidance. We will persist in enhancing the quality and depth of our research.

**Question 3：**Please provide a more detailed data collection strategy and sampling methods employed for data collection. In addition, please also provide the time and duration for the data collection.

**Response:** We are deeply grateful for your comprehensive review and insightful feedback on our research. In response to your recommendations, we have elaborated on the data collection strategies and sampling methods employed in **Study 1 and Study 2**, as well as clarified the specific timeframe and duration of data collection.

Below, please find the revised content:

## Data collection

## Data for Study 1 were gathered between November 24 and December 8, 2022, employing a convenience sampling technique to disseminate paper-based questionnaires among visitors to the Two Rivers and Four Lakes Scenic Area. To enhance the representativeness of the sample, researchers strategically approached tourists across various locations and time periods within the scenic area, aiming to encompass a broader spectrum of visitor demographics. Prior to questionnaire distribution, researchers conducted preliminary screening inquiries to ascertain participants' status as tourists. Subsequently, a concise overview of the study's objectives and questionnaire contents was provided to potential respondents, followed by a request for their voluntary participation. Questionnaires were only distributed upon receiving an explicit indication of willingness to partake in the survey.

## Data collection

To examine the proposed model’s stability across destinations, a study was conducted among tourists visiting Xingping Ancient Town in Guilin, China, from February 24 to March 10, 2023. Paper questionnaires were administered to tourists within the Xingping Ancient Town scenic area through convenience sampling. To ensure a diverse sample, researchers approached tourists in different sections of the scenic area, at various exits, and during different time periods, thereby encompassing a broader spectrum of tourist demographics. Prior to distributing the questionnaires, researchers conducted preliminary screening to verify the participants' status as tourists. Subsequently, the researchers provided a concise overview of the study's objectives and the questionnaire's content, and sought the consent of potential participants before proceeding with the distribution of the questionnaires.

We are deeply grateful for your invaluable guidance. We will persistently endeavor to enhance the quality and rigor of our research.

**Question 4：**It would be important to consider the heterogeneity of residents in the discussion as well. For example, different people would tend to make very different choices or be more sensitive to some specific factors. There is an recent example discussing this: “Determinants and mechanisms driving energy-saving behaviours of long-stay hotel guests: Comparison of leisure, business and extended-stay residential cases”.

**Response:** We wish to convey our deepest appreciation for your valuable insights. In this study, our primary objective was to validate the robustness of the research outcomes through the collection and analysis of data from two distinct types of tourist destinations. While the heterogeneity of residents was not the central focus, we did acknowledge its importance. However, due to constraints of scope and time, we were unable to delve deeply into the characteristics and variations among residents across different tourist destinations, nor could we explore how these differences specifically impact tourists' experiences and well-being. We recognize the critical importance of such investigations for improving the appeal of tourist destinations and enhancing tourist satisfaction.

One of the limitations of this study, as discussed in the section on **limitations and future research directions**, is the need for further exploration of this topic. Through more detailed research designs and methodologies, it will be possible to elucidate the specific mechanisms through which resident heterogeneity impacts tourists' SWB. This will enable us to provide more targeted recommendations for the planning and management of tourist destinations.

Regarding the article you mentioned, it is indeed of high quality and worthy of both study and emulation. We have cited it in this article. The following section contains additional content:

Thirdly, this study selected two distinct types of case sites for investigation, with a primary aim to validate the robustness of the research findings through rigorous testing. However, it is acknowledged that different individuals frequently exhibit varying preferences or heightened sensitivity to particular factors(77). The present study overlooked the heterogeneity of residents in its analysis. Consequently, future research should prioritize examining the heterogeneity of residents to gain a deeper understanding and more nuanced analysis of the variations in tourism choices and behaviors across different resident groups.

1. *Wang Q C, Lou Y N, Liu X, et al. Determinants and mechanisms driving energy-saving behaviours of long-stay hotel guests: Comparison of leisure, business and extended-stay residential cases[J]. Energy Reports, 2023, 9: 1354-1365.*

Thank you again for your valuable advice. Your guidance has provided new ideas and directions for our research.

**Question 5：**An new conclusion section rather than “discussion and conclusion” would be more helpful, as the current version would be a little bit long to summairse the core ideas of this research.

**Response:** We are deeply grateful for your invaluable feedback. We acknowledge that dividing the "Discussion and Conclusion" section into separate "Conclusion" and "Discussion" sections will enhance the clarity of the study's main points. Consequently, we have restructured the article and composed distinct "Conclusion" and "Discussion" sections. The revised content is as follows:

## Discussion

This study selected tourists from two distinct tourist destinations as research subjects to explore the intrinsic relationships between HGI, PA, SWB, and TTB through the construction of a conceptual model. By systematically collecting and analyzing feedback from tourists at these destinations, this study has not only enriched the body of knowledge on tolerance behavior but also expanded the existing research scope.

Firstly, we have confirmed that HGI exerts a significant positive influence on TTB. The HGI is considered a key driver in promoting the long-term sustainable development of tourist destinations**(12)**. Prior research has predominantly examined HGI as a prerequisite or moderating variable influencing residents' behaviors **(24)**, with a focus on its effects on tourists' green behaviors**(21)** and environmentally responsible actions **(22)**. Nevertheless, the mechanisms through which HGI impacts TTB are more intricate and multifaceted, and existing research remains inadequate. Tolerant behavior not only facilitates tourists' internal harmony but also offers valuable opportunities for service providers to rectify damaged customer relationships resulting from service failures **(76)**.

Secondly, our research indicates that PA serves as a mediating factor between HGI TTB. PA signifies the evolving emotional connection between tourists and tourist destinations **(31)**, while HGI enhances this attachment. This finding aligns with prior studies demonstrating that HGI significantly influence tourists' cognitive and emotional responses**(33)**. Furthermore, the strengthening of the human-place emotional bond positively impacts TTB, corroborating existing research that tourists' emotional attachment and sense of identity shape their attitudes toward tourist destinations, thereby influencing their behaviors and decisions**(39)**. We propose that in the context of open tourist destinations, where tourists have easy access to communication with local residents, this interaction facilitates the reinforcement of the emotional bond between tourists and destinations, leading to greater willingness to tolerate imperfections or negative aspects of the tourist experience.

Thirdly, we have confirmed that SWB acts as a partial mediator between HGI and TTB. SWB represents individuals' cognitive assessments of their own life satisfaction **(42)**. Prior research predominantly concentrated on the influence of HGI on residents' well-being**(50, 51)**, whereas this study broadens the scope to include tourists at tourism destinations, offering a novel perspective on the positive effects of HGI on tourists. Our findings suggest that within the tourism context, HGI can enhance tourists' SWB, and when tourists experience elevated levels of well-being at their destinations, they are more inclined to exhibit understanding and tolerance. This aligns with the theory of emotion regulation, which posits that an individual's emotional state substantially impacts their behavior **(54)**. Specifically, the communication and interaction between tourists and local residents at tourism destinations not only influence the quality of tourists' travel experiences but also profoundly affect their SWB. Tourists with higher SWB levels are more likely to adopt a positive attitude towards tourism-related unfavorable situations.

## Conclusion

In conclusion, despite the necessity for further replication and extension, this study has explored the impact of HGI on TTB for tourist destinations through the lenses of PA and SWB. Two empirical findings indicate that HGI can foster tourists' emotional connection to tourist destinations and enhance their overall well-being, while simultaneously increasing inclusivity towards tourist attractions. This research suggests that PA and SWB serve as potential mechanisms linking HGI to TTB. Moreover, tourists' PA and SWB function as a sequential mediator in the relationship between HGI and TTB. The mediator and multiple mediator analyses support the plausibility of the following pathway: (1) HGI → PA → TTB; (2) HGI → SWB → TTB; and (3) HGI → PA → SWB → TTB.

For your convenience, we have highlighted the revised sections in blue within the document. We are deeply grateful for your valuable feedback and support. Should there be any areas for enhancement, we would be most appreciative of your suggestions and will strive to make further improvements. Once more, we thank you for your diligent efforts and professional guidance.
