# Supplementary material for: Does host-guest interaction promote tolerance behavior? The mediating role of place attachment and subjective well-being
Source: PLoS One. 2025 May 28;20(5):e0323355. doi: 10.1371/journal.pone.0323355 (PMC12118835; doi:10.1371/journal.pone.0323355)
Supplement: Attachment — Submitted filename: Response to Reviewer.docx [file pone.0323355.s004.docx]

**Response Letter**

**Dear Editor:**

First of all, we would like to extend our sincere gratitude to the reviewer for their insightful comments and constructive suggestions on our previously submitted manuscript. Their feedback has been invaluable, not only significantly enhancing the quality of my research but also elevating the overall standard of the manuscript.
 Following a thorough revision process, we have meticulously addressed each of the reviewers' recommendations, resulting in comprehensive improvements to the manuscript. These enhancements have substantially strengthened the content, logic, and clarity of expression, bringing the manuscript into closer alignment with the publication standards of your esteemed journal. Should there be any further areas requiring refinement, we would greatly appreciate your guidance and will endeavor to make additional improvements accordingly.
 Once again, we express our profound appreciation for your diligent work and professional expertise. We look forward to your valuable response..

Yours sincerely,

Ke Wu

**Reviewer: 1**

We would like to extend our sincere appreciation to you for dedicating your valuable time to evaluate our manuscript. The constructive feedback provided has significantly enhanced the quality of our submission. Herein, we wish to convey our deepest gratitude.

With respect to all the points raised, we will address each one sequentially.

**Question 1：**First, the theoretical model requires further development. The application of the SOR model to this context, particularly the role of HGI as the stimulus, needs to be more clearly articulated and justified. The choice of PA and SWB as mediators, and their proposed chain relationship, also needs stronger theoretical support.

**Response:** We are most grateful for your thorough review of our research and for offering valuable feedback. Specifically, concerning the suggestion that the theoretical model requires further development, particularly in clearly explaining and substantiating the application of the SOR (Stimulus-Organism-Response) model and the role of host-guest interaction (HGI) as a stimulus factor, we have made the following revisions. In the "**Theoretical Basis and Hypothesis**" section, we have expanded on the foundational principles of SOR theory and its applications across various domains. We have meticulously analyzed the mechanism by which interaction functions as a stimulus factor within the SOR model. Through an extensive review of relevant literature, we have identified that interaction significantly influences an individual's psychological state (Organism), thereby guiding specific behavioral responses (Response). This insight provides a robust theoretical foundation for our subsequent research and enables us to construct and validate the applicability of the SOR model in the context of host-guest interaction more rigorously.

**SOR Theory**

In 1974, Mehrabian and Russell expanded upon the S-O-R theory within the framework of environmental psychology.They posited that various elements of the external environment serve as stimulus factors(S), which influence an individual's cognitive, emotional, and physiological states(O), ultimately shaping the individual's attitudes and behavioral responses(R)(16). The organism, representing the internal emotional and cognitive condition of tourists(17), acts as a mediator between external environmental stimuli and tourists' behavioral responses(16).

The validity of the SOR theory has been extensively empirically tested across various domains. Existing research demonstrates that emotional interaction, characterized by familiarity and intimacy as environmental stimuli, plays a pivotal role in influencing users' purchase intention processes(18). Interactions among users positively impact user perception, facilitating a deeper understanding of the product and enhancing user behavior(19). In marketing, leveraging social media can augment customers' SWB, thereby fostering increased brand loyalty(20). Within the tourism sector, positive HGI, destination image, trust, and attitudes positively promote intentions to support tourism(21). Furthermore, natural empathy and perceived environmental responsibility mediate the relationship between online interactions and consumers' intentions toward low-carbon tourism behaviors(22).

Nonetheless, there remains a paucity of research examining the influence of HGI on TTB. Consequently, this study employs the SOR theory as its theoretical framework. Specifically, HGI serves as the stimulus factor, while place attachment (PA) and SWB function as organism factors reflecting tourists' internal emotional states. TTB is considered the behavioral response. This paper constructs a structural equation model to investigate not only the impact of HGI on TTB but also the mediating role of PA and SWB in this relationship.

Secondly, concerning the issue you raised regarding the selection of PA and SWB as mediator variables and the need for stronger theoretical support for their proposed chained relationship, we have provided specific enhancements and in-depth elaborations in the "**Theoretical Basis and Hypothesis**" section. Specifically, we have thoroughly introduced the core concepts of attachment theory and meticulously analyzed the relationships between PA and SWB, PA and behavior, and SWB and behavior within this theoretical framework. We have further elucidated how PA and SWB influence behavior through a sequential mediation process. These additions aim to provide readers with a more comprehensive and profound understanding of the theoretical underpinnings for our variable selection and the interaction mechanisms among these variables. Additionally, we have restructured and integrated the relevant literature and logical arguments in "**The mediating role of PA**" and "**The chain mediating effect of PA and SWB**" sections based on attachment theory to ensure that our research is theoretically rigorous and complete. We trust that these modifications will more accurately address your comments and enhance the theoretical depth and persuasiveness of our study. A brief summary of the supplementary content follows:

**Attachment Theory**

Attachment theory, which stems from the mother-infant bond theory, is a pivotal framework in the study of human relationships and provides a comprehensive structure for understanding the development of emotional bonds(23, 24). Attachment refers to the inherent human tendency to form emotional connections with specific entities, and this inclination can significantly influence an individual's judgment. Scholars generally agree that the attachment bond between individuals and places can foster a sense of security and well-being(25). Hong's research demonstrates a positive correlation between an individual's value attachment and SWB(26). Ujang further highlights that tourists' PA can enhance the generation of well-being(27). DiWu et al. found that the impact of tourists' PA on SWB varies across different dimensions(28).

Emotion, as a fundamental component of the human-environment relationship (29), occupies a pivotal role in attachment theory. This theory suggests that subjective emotional states significantly influence human behavioral choices(30). For example, Wang et al. utilized attachment theory to conduct an in-depth analysis of the social attachment factors underlying community members' purchasing behaviors in business activities(31). Shallcross et al. examined the potential link between attachment orientation and the propensity to share positive news within the framework of attachment theory(32). Cohn posited that well-being serves as an external manifestation of emotional states and can directly impact behavioral outcomes(33). Individuals experiencing positive emotions tend to exhibit higher levels of well-being, which in turn positively reinforces behavioral outcomes(33). Furthermore, research indicates that SWB mediates the relationship between PA and pro-environmental behavior(34). Consequently, this paper hypothesizes that PA not only influences SWB but also that both PA and SWB may function as mediating variables in the process by which HGI affects TTB.

**The mediating role of PA**

Attachment theory posits that PA, defined as a deep emotional bond between individuals and their environments, is a critical psychological construct that substantially influences tourists' behaviors and decision-making processes(54). Wang et al. utilized attachment theory to elucidate the motivations behind purchasing behaviors among community members in commercial contexts and evaluated the influence of social attachment on such behaviors(31); Shallcross et al. explored the correlation between attachment orientation and the propensity to share positive news within the framework of attachment theory(32). Moreover, prior research has demonstrated that PA exerts a positive and significant effect on tourists' environmental responsibility behaviors(55), behavioral intentions(56), and revisit intentions(57). Building on this body of literature, this study hypothesizes that PA can positively impact TTB.

**The chain mediating effect of PA and SWB**

Attachment theory is not only pertinent to interpersonal relationships(78), but it also serves as a framework for elucidating the emotional connection between individuals and their environments(79, 80). Empirical evidence demonstrates that tourists can develop a distinctive emotional attachment to their travel destinations, which significantly influences their SWB(27, 28). More specifically, the emotional bond formed between an individual and a particular region can provide psychological security and a sense of social belonging(30), thereby effectively enhancing SWB(81, 82). Furthermore, research indicates that PA is linked to both physical and mental health(83, 84) and can foster positive emotional and behavioral responses(85, 86). Based on these findings, this study proposes the following hypothesis:

**H8).**PA has a significant positive effect on SWB.

In prior research, PA and SWB have been extensively validated as critical mediating factors. Specifically, PA mediates the relationship between local social identity and well-being(87), while community attachment serves as a mediator between community environmental perception and residents' SWB(88). Furthermore, Lin highlighted that SWB functions as an intermediary between PA and pro-environmental behavior(34). Wang demonstrated that PA and SWB exert a sequential mediating effect on the influence of environmental cognition on the loyalty of rural homestay summer vacationers(89). Drawing on SOR theory, attachment theory, and the aforementioned hypotheses, we posit that HGI, as an external stimulus, can influence TTB by enhancing their PA and SWB. Based on these theoretical frameworks and empirical findings, this study proposes the following hypothesis:

**H9).**PA and SWB play a chain-mediated role between HGI and TTB.

**Question 2:** Second, the measurement of HGI relies solely on self-reported data, which is potentially problematic. Exploring more objective or nuanced measures of HGI would strengthen the study's findings.

**Response:** We are deeply appreciative of your meticulous review of our research and the invaluable suggestions provided. With regard to your concern that the measurement of HGI is solely reliant on self-reported data, we offer the following response:

The methodology for measuring HGI is grounded in established approaches from prior studies (e.g., **Stylidis et al., 2020; Qu et al., 2024**), which provide a robust theoretical foundation and methodological support for our investigation. To address potential common method bias, we have included additional test results in the manuscript, specifically employing Harman's single-factor test. The findings indicate that common method bias has not substantially influenced our research outcomes. However, we acknowledge the inherent limitations of self-reported data and recognize this as a constraint of our current study. Consequently, in the "**Limitations and future research directions**" section, we have highlighted this issue and proposed exploring more objective and comprehensive data collection methods (such as behavioral data, experimental data, or multi-source data) to supplement or replace self-reported data in future research. We believe these enhancements will significantly bolster the reliability and scientific rigor of our research findings. The supplementary content is detailed below:

Secondly, this study utilizes self-reported data. This methodology may introduce biases due to participants' potential memory distortions when recalling past experiences or behaviors, which could compromise the accuracy of the data. Consequently, future research should explore the incorporation of more objective and comprehensive data collection techniques (such as behavioral tracking, experimental observations, or multi-source validation) to either complement or substitute for self-reported data. This would significantly enhance the reliability and robustness of the research findings.

*Stylidis D. Exploring resident–tourist interaction and its impact on tourists’ destination image[J]. Journal of Travel Research, 2022, 61(1): 186-201.*

*Qu Y, Zhou Q, Cao L. How do positive host-guest interactions in tourism alter the indicators of tourists’ general attachment styles? A moderated mediation model[J]. Tourism Management, 2024, 105: 104937.*

**Question 3:**Third, the rationale for conducting two separate studies is not entirely convincing. A more integrated approach, or a clearer articulation of the unique contribution of each study, would be beneficial.

**Response:** We express our sincere gratitude for your thorough review of our research and the invaluable suggestions offered.

In this study, the primary objective of conducting two independent studies is to validate the robustness of our research findings. By systematically collecting and analyzing data from both natural heritage sites and cultural heritage sites, we can comprehensively examine the relationships among variables. A literature review reveals that other scholars have similarly employed this method to verify the robustness of their conclusions. For example, **Su** conducted surveys of tourists in Xiamen and Yuelu Mountain to investigate the relationship between service quality and subjective well-being, providing valuable methodological support for our study. Rather than discussing the results of each study separately, we opted for a comprehensive analysis to enhance the generalizability and robustness of our conclusions. The findings from both studies indicate consistent relationships among variables across different types of scenic spots, further supporting our research outcomes. Additionally, to avoid excessive length, we chose to emphasize the commonalities and unique significance of the two studies in the integrated discussion.

We trust that our explanation will alleviate any concerns you may have and we anticipate your valuable feedback and guidance on our research with keen interest.

*He X, Su L, Swanson S R. The service quality to subjective well-being of Chinese tourists connection: A model with replications[J]. Current Issues in Tourism, 2020, 23(16): 2076-2092.*

For your reference, we have highlighted the revised sections in blue. We sincerely appreciate your valuable feedback and suggestions. We remain committed to continuously enhancing the quality of our research. Should you have any further recommendations for improvement, we would be most grateful to receive them and will endeavor to implement enhancements accordingly. Thank you once again for your diligent efforts and professional guidance.
